# Gut microbes as medical signature for the effectiveness of immunotherapy in patients with advanced non‐small cell lung cancer

**DOI:** 10.1002/agm2.12292

**Published:** 2024-02-22

**Authors:** Adane Adugna, Yalew Muche, Mohammed Jemal, Samuel Derbie Habtegiorgis, Habtamu Belew, Gashaw Azanaw Amare

**Affiliations:** ^1^ Medical Laboratory Sciences, College of Health Sciences Debre Markos University Debre Markos Ethiopia; ^2^ Department of Biomedical Sciences, School of Medicine, College of Health Sciences Debre Markos University Debre Markos Ethiopia; ^3^ Department of Public Health, College of Health Sciences Debre Markos University Debre Markos Ethiopia

**Keywords:** gut microbes, immunotherapy, medical signatures, non‐small cell lung cancer

## Abstract

Lung cancer (LC) is the most common cause of cancer‐related death worldwide and poses a severe threat to public health. Immunotherapy with checkpoint blockers has improved the outlook for advanced non‐small cell lung cancer (NSCLC) therapy. For the treatment of patients with advanced NSCLC, antibodies such as anti‐programmed death 1 (anti‐PD1), anti‐programmed death ligand 1 (anti‐PD‐L1), and anti‐cytotoxic T lymphocyte‐associated antigen 4 (anti‐CTLA‐4) are of paramount importance. Anti‐PD‐1 and anti‐PD‐L1 monoclonal antibody therapies are used to block the PD‐1/PD‐L1 pathway and identify cancerous cells to the body's defenses. Antibodies directed against CTLA‐4 (anti‐CTLA‐4) have also been shown to improve survival rates in patients with NSCLC. Currently, other immunotherapy approaches like neoadjuvant immune checkpoint inhibitors (NAICIs) and chimeric antigen receptor T‐cell (CAR‐T) therapies are applied in NSCLC patients. NAICIs are used for resectable and early stage NSCLC and CAR‐T is used to find more useful epitope sites for lung tumors and destroy cancer cells. A patient's gut microbiota might influence how their immune system reacts to NSCLC immunotherapy. The majority of intestinal microbes stimulate helper/cytotoxic T cells, induce natural killer (NK) cells, activate various toll‐like receptors (TLR), build up cluster of differentiation 8 (CD8), increase PD‐1 production, and attract chemokine receptors towards cancer cells. Thus, they serve as immune inducers in NSCLC immunotherapy. Nonetheless, certain bacteria can function as immune suppressors by inhibiting DC proliferation, stopping CD28 trafficking, restoring CD80/CD86, increasing immunological tolerance, and upsetting Th17 cells. Therefore, they are prevalent in non‐responders with NSCLC immunotherapy.

## Graphical abstract

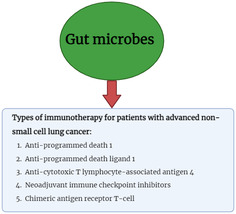

## 1 INTRODUCTION

Globally, lung cancer (LC) is a serious issue for public health and the leading cause of cancer‐related mortality alongside 1.8 million fatalities projected in each year. The major type of lung cancer is advanced non‐small cell lung cancer (NSCLC) and accounts for 85% of all cases. The remaining 15% can be attributed to small cell lung cancer (SCLC).^1^ As the clinical stage advances, the patient rate of survival decreases from over 80% in the initial phase to roughly 5% in the fourth stage. Approximately 70% of those suffering from NSCLC possess a severe stage, whether locally or through organ metastases.^2^, ^3^

Nowadays, cancer immunotherapy including NSCLC immunotherapy is growing at a rapid pace.^4^ Anti‐programmed death 1 (anti‐PD‐1), anti‐programmed death ligand 1 (anti‐PD‐L1), anti‐cytotoxic T lymphocyte‐associated antigen 4 (anti‐CTLA‐4), neoadjuvant immune checkpoint inhibitors (NAICIs), and chimeric antigen receptor‐modified T (CAR‐T) are among the current immunotherapy for patients with NSCLC. Rebooting the cancer's immunological loop and reestablishing the body's native defenses are the two main ways that cancer immunotherapy regulates and eradicates cancers.^5^

The composition of the patient's microbiome has been thoroughly investigated for its association with NSCLC immunotherapy.^6^ Investigations using fecal microbiome grafts from patients with cancer to mice have shown that the gut microbiota affects how the body reacts to medications called checkpoint inhibitors (CPIs).^7^ Immune checkpoint inhibitors (ICIs) such as PD‐1 and CTLA‐4 are the primary immunotherapy approaches presently administered for patients with NSCLC.^8^

Checkpoint blockers, a class of medications used in immunotherapy, disrupt molecules like PD‐L1 that prevent immune cells from identifying and eliminating tumor cells. It is possible for immunological cells to identify and combat lung cancer after the molecules are disabled. Human reaction to treatment with anti‐PD‐1 or anti‐PD‐L1 checkpoint medications is linked with intestinal microbes.^9^

The fragile equilibrium among tolerance to immunity and pulmonary stimulation can be destroyed by intestinal dysbiosis, which can also result in tumor‐causing inflammation from an overreaction to the immune system or lower therapeutic efficacy from a compromised immune monitoring system.^10^ The microbiota of the intestines is believed to be a unique indicator for lung cancer individuals' immunotherapy responsiveness and adverse reactions.^11^ As a result, the present developments in the microbiological markers of NSCLC immunotherapy will be discussed in this review to figure out its efficacy.

## 2 GUT MICROBES AS MEDICAL SIGNATURE FOR THE EFFECTIVENESS PD‐1/PD‐L1 THERAPY IN NSCLC PATIENTS

The latest and most advanced treatment for patients who have severe NSCLC is immunotherapy with anti‐PD‐1 and anti‐PD‐L1 antibodies that are monoclonal. Both solo and combined therapy may extend the individual's life with minimal adverse effects.^12^ ICIs are commonly utilized in the treatment of progressing or stage IV non‐small‐cell lung cancer.^13^

**FIGURE 1:**
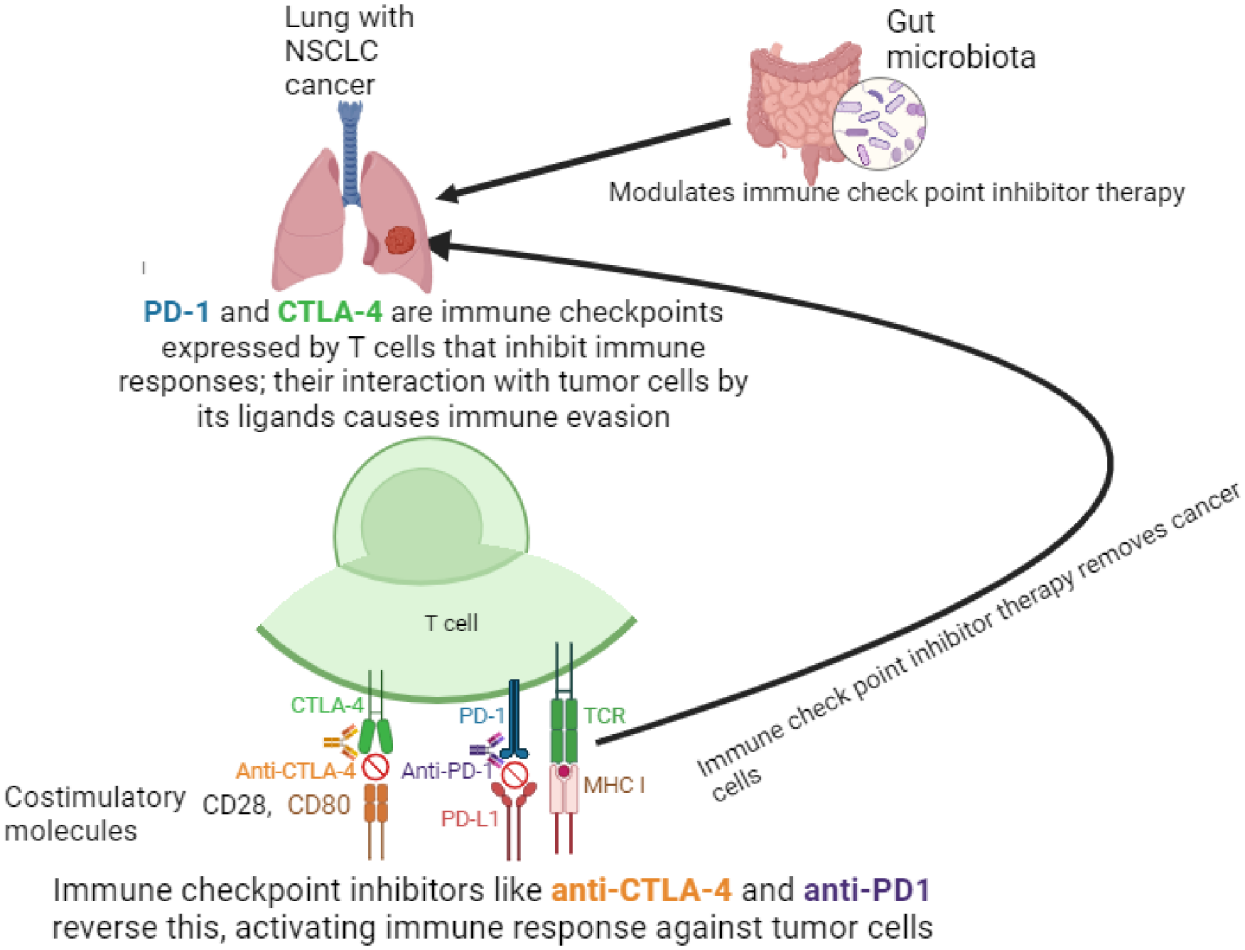
Immune checkpoint blockades (ICBs) therapy for NSCLC patients.

Cancer cells are more vulnerable to T cell identification and destruction when their inhibitory pathways are inhibited. We call these types of antibodies anti‐CTLA‐4 and anti‐PD‐1/PD‐L1, immune checkpoint blockades (ICBs).^14^ Cancerous cells employ the chemical PD‐L1 in order to conceal from the host's immune system by generating it on their outermost layer. When PD‐L1 attaches to the PD‐1 receptors on T lymphocytes, the body's immune system ceases to attack the malignant cells, preventing the tumor cells from being destroyed.^15^ Anti‐PD‐1 and anti‐PD‐L1 monoclonal antibody therapies are used in immunotherapy to inhibit the PD‐1/PD‐L1 route, which makes cancerous cells identifiable to the body's defenses and enables it to trigger immune system functions that eventually kill malignant cells.^16^ The sole determinant of eligibility for immunotherapy in patients with NSCLC, is the existence of PD‐L1 molecule expression on cancer cells as identified by immunohistochemically^17^ (Figure 1).

The goal of presently licensed cancer immunology medications for the management of pulmonary cancer is to block immunological checkpoints, including cytotoxic T lymphocyte‐associated antigen 4 (CTLA‐4), PD ligand‐1 (PD‐L1), and programmed death protein‐1 (PD‐1).^18^ The two most well‐known gatekeeper mechanisms, CTLA‐4 and PD‐1/PD‐L1, limit T‐cell function in different manners. While PD‐1 controls later effector T‐cell activation within tissue and malignancies, CTLA‐4 modulates T‐cell function at the outset. Since their widespread development, ICBs aimed at PD‐1, CTLA‐4, and PD‐L1 have demonstrated favorable outcomes in the treatment of NSCLC.^19^

Over half of those diagnosed with severe non‐small cell lung cancer respond to ICIs when they are given as initial therapy to individuals with PD‐L1 mutations on over 50% of the cancer cells. Even in initially responding, advanced non‐small cell lung cancer patients receiving immunotherapy, developed resistance may appear.^20^

Merely 20% of NSCLC patients have persistent cancer or appropriately react to immunotherapy, and a small proportion of patients get long‐lasting improvements.^21^ Previous study has demonstrated that the microbiota of the gut may affect the effectiveness of ICIs. Commensal microbiome proteins have the ability to cross the intestinal walls and trigger the generation of cytokines and interferons, activate T cells, and activate Toll‐like receptors via the gut‐lung nexus.^22^

Molecular replication is the term for this process, in which tumor neoantigen‐like receptors generated by gut‐dwelling microbial species as part of their endogenous DNA replication processes can mimic cancer neoantigens, stimulating self‐reactive T‐cells and strong immune response against tumors.^23^ Researchers have recently demonstrated that Bacteroidales play a major part in the stimulation of the immune system linked to ICIs.^24^

Gut microbiome linkage metagenomic indicators may have relationships with the immune system surrounding the cancerous cells.^25^ ICI cytotoxicity was also linked to disturbed microbiota in the intestines.^26^

Several species of Streptococcus have a strong correlation with reduced ICI effectiveness, while the existence of *Bifidobacterium*, *Alistipes*, *Tannerellaceae*, and *Barnesiella* is associated with improved results. Mice infected with *Bifidobacterium* showed enhanced dendritic cell activity, and a buildup of CD8+ T lymphocytes in the cancerous milieu and these outcomes were primarily brought about by the maturation stimulation of dendritic cells (DCs), which improved the cellular activity of CD8+ T lymphocytes that are unique to cancers. According to the machine learning method, metabolic processes are the most reliable source of information to forecast the PD‐L1 phenotypes. *Methanobrevibacter smithii*, the sole species substantially prominent in PD‐L1‐low individuals with respect to PD‐L1 position, has an adverse connection with taxa related to PD‐L1‐high circumstance, namely *Lachnoclostridium* sp. and *Ruminococcus gnavus*.^27^, ^28^, ^29^

According to the previous studies, the existence of *Akkermansia mucinifila* and *Faecalibacterium prausnitzii* bacteria in the gut served as marker for the response of immunotherapy in patients with lung cancer. It has been suggested that if these bacteria are present in the intestine, patients with NSCLC who receive immune checkpoint inhibitors (ICIs) react more effectively to therapy.^7^, ^30^

Utilizing multicolored flow cytology to investigate immune system responses, it was found that individuals who had an elevated gut microbiota variability density were more likely to have distinct repertoire CD8+ T cell and natural killer cell (NK) subgroups in the peripheral region in the aftermath of anti‐PD‐1 medication.^28^

The colonization of *A. muciniphila* in the intestines showed successful ICI treatment outcomes in NSCLC patients. Patients with advanced NSCLC receiving anti‐PD‐1/anti‐PD‐L1 therapy in the second line have a favorable association between the gut's *Akkermansiaceae* and the disease's stability and immunotherapy response. This finding implies that *A. muciniphila* in the gastrointestinal tracts of individuals receiving anti‐PD‐1 antibody treatment aids in triggering the body's defenses to successfully attack cancer cells.^30^

Toll‐like receptors (TLR) 2 and TLR4 were activated by proteins that were made by *A. mucinifila*, which resulted in the release of particular cytokines, primarily high quantities of interleukin 10 (IL‐10) and interleukin 12 (IL‐12) and enhance the generation of interferon‐gamma (IFN‐ γ).^7^, ^31^ *B. breve* and *B. longum* are closely connected and have a suitable response to anti‐PD‐1 treatment.^32^

According to a mouse cancer paradigm, *A. muciniphila* taken orally following transplantation of fecal microbiota in mice not responding to therapy restored the efficacy of PD‐1 inhibition in an interleukin‐12‐dependent fashion by attracting CCR9 (C‐C chemokine receptor type 9) + CXCR3 (C‐X‐C chemokine receptor 3) + CD4 + T cells into cancer.^7^

It was also recently discovered that bacteria belonging to *Ruminoccocaceae*, *Barnesiellaceae*, *Enterococcceae*, *Escherichia*, *Shigella*, *Olsenella*, and *Rikenellaceae* may play a significant role as immunotherapy accelerators.^33^, ^34^

Certain bacterial like *Parabacteroides diastonis*, *Proteobacteria*, *Prevotellaceae*, and *Fusobacteria* are linked to ICI opposition and prevalent in non‐responder patients, while *Lactobacillus* and *Blautia* spp. have been frequently related to a favorable reaction. These important findings suggest that assessing the gut microbiome's promise as an immunotherapy indicator requires characterizing the microbiota of responders relative to non‐responders.^35^ Moreover, individuals with elevated levels of bacteria such as *Clostridium*, *Syntrophococcal*, and *Lactobacillus* are responders to anti‐PD‐1 monotherapy. In contrast, patients with high concentrations of *Sutterella* and *Bilophila* are non‐responders.^36^ Furthermore, it is important to note that a lower prognosis for individuals with NSCLC receiving anti‐PD‐1 medication has also been associated with Helicobacter pylori seropositivity.^37^

Furthermore, the phylum‐level makeup of fungi was also examined in the previous study in patients with NSCLC after received immunotherapy. The phylum *Ascomycota* topped in responders and non‐responders, followed by *Firmicutes*, *Basidiomycota*, *Chytridiomycota*, *Microsporidia*, *Mucoromycota*, *Candida*, *Malassezia*, and *Neosartorya*. In addition, *Aspergillus terreus* and *Verticillium longisporum* and *Aspergillus homomorphous* and *Lachancea mirantina* exhibited the greatest positive relationships among respondents, while the strongest inverse relationships were observed between *Enterocytozoon bieneusi* and *Debaryomyces hansenii* and *Debaryomyces hansenii*, *Lachancea mirantina*, and *Schizosaccharomyces octosporus*.^38^, ^39^, ^40^, ^41^

On the other hand, a highly diverse microbiome in the intestines promotes the development of T‐helper 1 (Th1) and M1 macrophages, the stimulation of helper/cytotoxic T cells, and the increase of PD‐1 production on lymphocytes.^42^ Blood tests from individuals who had elevated gut variety in the microbiome showed a rise in memory T and NK cell signatures.^28^ An overview of the gut microbiomes that has been linked to the response to PD‐1/PD‐L1 blockade therapy in patients with NSCLC are compiled into Table 1.

**TABLE 1:** Gut microbiomes as medical signatures for NSCLC patients with PD‐1/PD‐L 1 inhibitor therapy.

Author	Year of study	Types of gut microbes	Subject	Immunomodulation mechanisms	Ref.
Routy et al., Grenda et al	2018, 2022	*Akkermansiacea muciniphila*, *Tannerellaceae*, *Escherichia*, *Shigella*, *Olsenella*, and *Rikenellaceae*.	Human, Mouse	They boost the host immunity during NSCLC immunotherapy by increasing Th1 lymphocytes and in IL‐12‐dependent manner, boosting the amount of IFN‐γ, and attracting CCR9 + CXCR3 + CD4 + T cells towards cancer cells.	7, 29
Jin et al., Zhang, M., Liu, J., Xia, Q.	2019, 2023	Alistipes, putredinis, Bifidobacterium longum, Prevotella, and *copri*	Human, Mouse	Via rising distal natural killer (NK) cells and inducing cancer‐specific CD8+ T cells with specific memories, lowering pro‐inflammatory cytokines including TNF and IL‐17, and through boosting the quantity of Th17 lymphocytes and FOXP3 in circulation within cancer mattresses.	28, 43
McLean et al.	2022	*Parabacteroides diastonis*, *Fusobacterium* spp., *Proteobacteria*, *Prevotellaceae*, *Fusobacteria*, and *H. pylori*.	Human, Mouse	They resist the induction of anti‐PD‐1/anti‐PD‐L1 associated immunresponse by generating harmful or carcinogenic substances, disrupting Th17 cell activity, and stimulating Tregs	35
Liu, T., Xiong, Q., Li, L., Hu, Y.	2019	*Clostridium*, *Syntrophococcal*, and *Lactobacillus*.	Human	They help DC development and act as an amplifier of immunity against cancer.	36

Abbreviations: CCR9, C‐C chemokine receptor type 9; CD4, Cluster of differentiation 4; CD8, Clusters of differentiation 8; CXCR3, C‐X‐C chemokine receptor 3; DCs, Dendritic cells; FOXP3, Forkhead Box Protein P3; IFN‐γ, Interferon gamma; IL‐12, Interleukin 12; IL‐17, Interleukin 17; NK, Natural killer; PD‐1, Programmed Death 1; PD‐L1, Programmed Death Ligand 1; spp., species; Th1, T‐helper 1; TNF, Tissue necrosis factor; Tregs, Regulatory T‐cells.

## 3 GUT MICROBES AS MEDICAL SIGNATURE FOR THE EFFECTIVENESS CTLA‐4 THERAPY IN NSCLC PATIENTS

The protein known as CTLA‐4 is present on the outermost layer of T cells and, when activated, suppresses T cells that have been triggered, thereby stopping the immunological response. But a variety of tumor cell types have the ability to stimulate CTLA‐4 in the tumor's microenvironment, which enables the tumor cells to avoid being recognized and eliminated by the immune system by early suppressing T lymphocytes.^44^

The activated T cell is rendered inert by the co‐inhibitory receptor CTLA‐4, which binds B7 more strongly than cluster of differentiation 28 (CD28). The immune response can be stopped, permitting the CTLA‐4 contact. Furthermore, T cells display a protein called PD‐1, which functions analogous to CTLA‐4 in ending a T cell response by adhering to PD‐L1 or PD‐L2 of additional cells like cancer cells.^45^ It has been demonstrated that antibodies targeting CTLA‐4 increase survival rates in a large number of malignant tumor individuals, especially those with NSCLC.^7^

The function of regulatory T lymphocytes (Tregs) is to suppress lymphocytes that are cytotoxic (CTLs) in order to avoid immunological overstimulation and the ensuing inflammatory response. On their exterior, Tregs strongly express CTLA‐4, which quickly suppresses CTLs and prevents them from causing harm. Ipilimumab, a kind of monoclonal antibody, binds to CTLA‐4 and reduces the suppression of CTLs' anti‐cancer activities.^46^

The makeup of the microbiome is also essential for the immunostimulatory consequences of CTLA‐4 inhibition. Anti‐CTLA‐4 antibody treatment causes dysbiosis in the stomach by lowering *Bacteroidales* and *Burkholderiales* bacteria and boosting the number of Clostridiales bacteria. In humans as well as mice, *Bacteroides* species influence the interleukin (IL) 12‐reliant Th1 immune system reaction, which aids in cancer management.^47^

In patients undergoing CTLA‐4‐based immunotherapy, a higher incidence of *Bacteroides fragilis*, *Burkholderia cepacia*, *Faecalibacterium genus*, *D. formicigenerans*, *C. aerofaciens*, *Eubacterium* spp., *Veillonella parvula*, *Klebsiella pneumoniae*, *Blautia* spp., *E. hirae*, *E. faecium*, *H. filiformis*, *Faecalibacterium prausnitzii*, and *Gemmiger formicilis* in the gut resulted in a more potent therapeutic impact while reducing unfavorable adverse reactions including diarrhea.^7^, ^44^

Further, mice that are germ free can induce the anti‐tumor immune response that has been observed in mice with a typical microbiota by oral treatment of *B. fragilis*, *B. thetaiotaomicron*, or a combination of *B. fragilis* and *B. cepacia*. These bacteria can stimulate the production of Th1 and enhance development of DCs. Therefore, they are prognostic factors for anti‐CTLA‐4 medication and result in reduction of cancers in mice model.^47^, ^48^

The capacity of these bacteria to cause DC development and ensuing production of IL‐12 by DCs located in the intestinal tract's propria membrane is probably the cause of the recovered reaction. Antigen presenting cells (APCs) that are called DCs prepare and deliver antigens to T lymphocytes so they can be eliminated. It's probable that all of the DCs participating in this reaction share the CD11b interface ligand. DCs' IL‐12 synthesis triggers Th1 cells, or T helper cells, to assist in executing the immune response to combat cancer. Moreover, the curative effect of the CTLA‐4 inhibition is also reestablished by adoptively delivering *B. fragilis*‐specific T lymphocytes into germ‐free mice. This implies that the ability of T cells to respond to anti‐CTLA‐4 immunoglobulin depends on the types of microbes. *B. fragilis* may produce polymer A, which is thought to increase the generation of IL‐10 and reduce inflammatory processes, possibly serving an immunoregulatory function in the CTLA‐4 axis.^14^

In contrast, microbes such as *Ruminococcus obeum*, *Roseburia intestinalis*, *Oribacterium sinus*, *Parasutterella excrementihominis*, *Scardovia wiggsiae*, and *Veillonella parvula* are found in non‐responders.^49^ Major intestinal microbiomes that have been associated to the response to CTLA‐4 blockade therapy in patients with NSCLC are summarized in Table 2.

**TABLE 2:** Gut micro‐biomes as medical signatures for NSCLC patients with CTLA‐4 inhibitor therapy.

Author	Year of study	Types of gut‐microbes	Subject	Immunomodulation mechanisms	Ref.
Vétizou et al., Roy, S., Trinchieri, G., Miller, P.L., Carson, T.L., Zhang, M., Liu, J., Xia, Q.	2015, 2017, 2020, 2023	*Bacteroides fragilis*, *Bacteroides thetaiotaomicron*, *Burkholderia cepacia*, *D. formicigenerans*, *C. aerofaciens*, *Eubacterium* spp., *Veillonella parvula*, *Klebsiella pneumoniae*, *Blautia* spp., *E. hirae*, *E. faecium*, *H. filiformis*, *Faecalibacterium prausnitzii*, and *Gemmiger formicilis*	Man, Mouse	They boost the Th1 response, encourage DC growth, promote CD8+ T cells counts, and decrease regulatory T‐cells (Tregs) in the cancer environment Increase the release of IL‐10 by induction of polysaccharide A, suppressing the expansion of tumors via declaring cancer cells' histone deacetylases, causing the immune system to become less inflammatory, and engaging in the presentation and processing of antigens All of which improved the effectiveness of anti‐CTLA‐4 medication	43, 44, 47, 48
Matson et al., Shaikh, F.Y., White, J.R., Gills, J.J., Hakozaki, T., Coutzac et al.	2018, 2020, 2021	*Ruminococcus obeum*, *Roseburia intestinalis*, *Oribacterium sinus*, *Parasutterella excrementihominis*, *Scardovia wiggsiae*, and *Veillonella parvula*	Human, Man	They are found in non‐responders individuals with CTLA‐4 inhibitor therapy and have suppressive activity through producing fatty acids with a short chain as microbial metabolites and reduce the growth of DC triggered by anti‐CTLA‐4, block the CD28 trafficking process, restore CD80/CD86, and boost immunological tolerance. These all result in resistance to immunotherapy against CTLA‐4	49, 50, 51

Abbreviations: CD8, Cluster of differentiation 8; CTLA‐4, Cytotoxic T lymphocyte‐associated antigen 4; DC, Dendritic cell; IL‐10, interleukin 10; spp., species; Th1, T‐helper 1.

## 4 GUT MICROBES AS MEDICAL SIGNATURE FOR THE RESPONSIVENESS OF NEOADJUVANT IMMUNE CHECKPOINT INHIBITORS (NAICIs) THERAPY IN INDIVIDUALS WITH REMOVABLE NSCLC

Immunotherapy has also been investigated as a neoadjuvant.^52^ According to various studies, neoadjuvant immunotherapy is a major component of multilingualism treatment for the initial stage as well as resectable NSCLC.^53^ For decreasing tumor mass before an operation, neoadjuvant medication offers potential benefits for people with lung cancer. In removable NSCLC, neoadjuvant immunotherapy was found to be effective as well as safe.^54^

Initially administering ICIs in the neoadjuvant context when the main cancer is still in existence could offer a wider range of responses from T cells than in the postoperative adjuvant phase following resection because immune checkpoint obstruction increases the stimulation of T cells upon antigen interaction.^55^, ^56^

Particularly, individuals taking neoadjuvant nivolumab in NSCLC were found to have median T‐cell replicas. This growth cells are the result of antigen dissemination following ICI therapy through stimulation and could also suggest the development of a novel T‐cell repertory that is better suited to support efficient anticancer immunotherapy.^57^

Eliminating the initial cancer also probably eliminates the possibility of T cell restlessness brought on by repeated interaction with antigens. In contrast to the equivalent adjuvant ICI, NAICI causes a considerably larger level of tumor antigen‐specific migrating T cells and creating an abundance of effector‐memory T‐cell responses that last for an extended period of time.^58^

The enduring viability of tumor antigen‐specific autologous T cells is observed among individuals who experienced long‐lasting advantages due to immune checkpoint blockade in NSCLC and in animal studies of successful neoadjuvant therapies.^59^

Mouse model treated with neoadjuvant anti‐PD‐1 lived lengthier than those treated with adjuvant anti‐PD‐1. Anti‐PD‐1 and anti‐CD137 together have more effectiveness than anti‐PD‐1 separately. According to the researchers, neoadjuvant immunotherapy boosts cancer‐specific CD8+ T‐cells in regions and blood vessels outside of the body. Consequently, following the initial cancer excision, neoadjuvant immunotherapy possessed a considerably higher capacity than postoperative immunotherapy to eliminate NSCLC and resulting in prolonged patients survival.^58^, ^60^ In a retrospective analysis, 5‐year over‐survival rates for neoadjuvant immunotherapy therapy and adjuvant immunotherapy therapy were 56.2% and 33.0%, respectively.^61^

Intestinal microbes including *Ruminococcaceae*, *Actinomycetales*, *Odoribacteraceae*, *Rikenellaceae Desulfovibrio*, *Lactobacillus*, and *Clostridium* have been found in the responder patients with NSCLC who received neoadjuvant immune checkpoint inhibition. However, microbes including *Raoultella* and *Agathobacter* have immunosuppressive role in patients with neoadjuvant immunotherapy.^62^, ^63^, ^64^, ^65^

## 5 GUT MICROBES AS MEDICAL SIGNATURE FOR THE RESPONSIVENESS OF CHIMERIC ANTIGEN RECEPTOR T‐CELL THERAPY IN NSCLC PATIENTS

Chimeric antigen receptor T‐cells (CAR‐T) are a new approach immunotherapy for NSCLC patents. In the realm of cancer therapy studies, CAR‐T cells have become known as an emerging field and area of emphasis as one of the developing approaches for cancer immunotherapy.^66^ Newly modified T cells or CAR‐T cells are used to reroute individuals' immune system reactions so that they can identify and destroy cancer cells that produce antigens linked to tumors.^67^ In CAR‐T cell treatments, blood from a patient is used to modify T cells in vitro. This alteration involves inserting a gene for a receptor known as chimeric antigen receptor (CAR) that aids in the T cells' attachment to a particular antigen on tumor cells. After that, the individual receives their CAR T cells again.^68^

Prior research has shown that CD28‐CAR‐T cells provide better in eradicating NSCLC cancerous cells. However, the paucity of antigens specific to the cancer, the hostile cancer microcosm, a lack of CAR‐T cell penetration into tissue from the cancer, and cancer protein egress make it difficult to target particular antigens in NSCLC using modified CAR‐T cells.^69^

To improve the tumor killing ability of T cells, the third‐generation CARs are based on the second generation by continuing to add co‐stimulatory molecules to CARs, and the fourth‐generation CARs, also known as T cells redirected for universal cytokine‐mediated killing have expanded the number of deoxyribonucleic acid (DNA)‐storing cytokines that help with immunotherapy of lung cancer by secreting a lot of inflammation‐causing cytokines like IL‐12, IL‐15, and granulocyte‐macrophage colony‐stimulating factor which further triggers and attract additional immune cells to fight the immune support specific cancer microcosm.^70^, ^71^, ^72^, ^73^

Innovative techniques to find more useful epitope sites for lung tumors are being researched in light of the potential of CAR‐T cell immunotherapy in preliminary models of the disease. For instance, C‐X‐C chemokine receptor type 4 (CXCR4) was found to be increased in lung tumor cells and tissues and was also found to be a useful treatment option for non‐small cell lung cancer.^74^

To combat NSCLC, transgenic CAR‐T cells that specifically target PD‐L1 and CD80/CD86 have been employed. Individuals suffering from advanced PD‐L1‐reactive NSCLC are also being studied for both safety and effectiveness using anti‐PD‐L1 CAR‐T‐cell treatment.^69^ Remarkable cytotoxic impacts targeting NSCLC cells were demonstrated by PD‐L1‐targeted CAR‐T cells both in culture and in tissue.^75^

There are numerous obstacles despite the fact that CAR‐T technology for lung cancer therapy is constantly improving. T cell exhaustion is one of the primary issues. Combining treatments aimed at exhaustion mechanisms will undoubtedly boost CAR‐T cell durability and effectiveness. In order to minimize adverse responses, attempts are underway to identify more precise antigens that are targeted for lung carcinoma cells. Additionally, CAR‐T cells have been frequently designed via advancements in genetic engineering, which allows for a higher proportion of CART cells to move to tumor regions and improve the anti‐lung tumor capacity.^67^

Such genetic alteration of T cells can happen by viral‐based transfer of genes techniques or non‐viral techniques including DNA‐based transposons, short palindromic repeats that are frequently spaced apart and direct electroporation of in vitro generated messenger ribonucleic acid (mRNA) to improve CAR‐T cells.^76^

**FIGURE 2:**
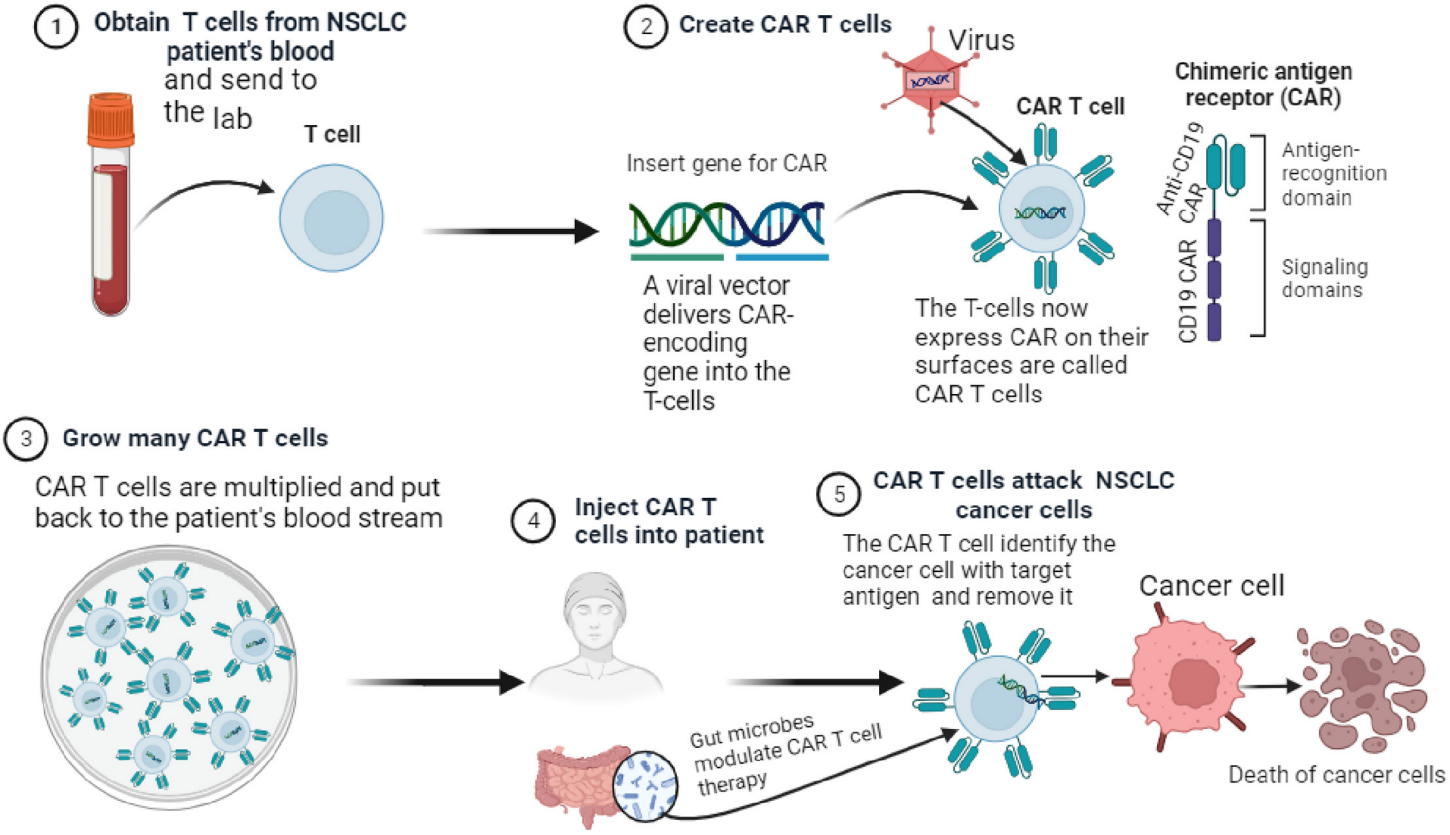
Mechanisms of chimeric antigen receptor T‐cell (CAR‐T) therapy for patients with NSCLC.

Individuals on CAR‐T therapy are subjected to a range of factor like gut flora. Gut microbiome can play a role in modulating the effectiveness of CAR‐T treatment. Research has shown that toll‐like receptor 2 (TLR2) generates co‐stimulatory impulses that enhance the synthesis of IL‐2, tumor necrosis factor alpha (TNFα), and IFNγ in both human and mouse CD8+ T cells. Additionally, these cues raise the proportion of polyfunctional T cells that can combat cancer cells. Surprisingly, in mouse experiments, activation of TLR2 in CARTs targeting CD19 has been linked to the growth, durability, and anti‐tumor efficacy of the corresponding CARTs. This is especially fascinating since lipoteichoic acid has been utilized by intestinal microbes like enterococci to activate TLR2 in immune cell populations^77^, ^78^ (Figure 2).

Furthermore, the gut microbiome's prevalence of specific species such as *Actinomyces*, *Prevotella*, *Collinsella*, *Herbaspirillum* spp., *Sphingomonadaceae*, *Aggregatibacter* spp., and *Cyanobacteria* contributes to the effectiveness of CAR‐T treatment. According to the latest research in vivo, microbial generated molecules can also be responsible for the microbiota regulation of CAR‐T therapy. Cancer size and mass were substantially decreased in a dermal animal model by CAR‐T treatment engineered to express a receptor tyrosine kinase‐like orphan 1 receptor, whereas immune effector cell‐associated neurotoxicity syndrome and cytokine release syndrome are among the negative impacts of dysbiosis caused by *Veillonella parvula* in a NSCLC mouse model.^79^, ^80^

Through stimulating inhibitory receptors and suppressing the generation of cytokines, additional microbial‐derived substances can also trigger CD8+ T‐cell depletion by affecting T cells' capacity to destroy cancer cells. This is achieved via signaling through aryl hydrocarbon receptors.^81^, ^82^

## 6 CONCLUSIONS

Despite the fact that immunotherapy is starting to show progress in treating advanced NSCLC, it remains imperative from a medical point of view to more accurately identify individuals who will respond to therapy. The effectiveness of advanced NSCLC immunotherapy and vital medical results are influenced by the gut flora. Patients with advanced NSCLC benefit most from ICIs when their intestinal flora variety is higher. As a result, intestinal microbes can serve as biomarker for both responders and non‐responders.

## 7 RECOMMENDATIONS AND FUTURE PERSPECTIVES

We recommend academic, scientific, and research communities to perform advanced original molecular research at genomic level to characterize gut microbiota as medical biomarkers in patients with advanced non‐small cell lung cancers and recognize the prognosis and anti‐tumor efficacy of immunotherapy. It is also better to investigate several metabolites and molecules produced by gut microbiomes to understand their immunomodulation role in advanced non‐small cell lung cancer patients.

## AUTHOR CONTRIBUTIONS

AA: Involved in the conception, manuscript draft, design, manuscript writing‐up, manuscript editing, and validation. YM: Involved in manuscript writing‐up. MJ: Involved in language editing and manuscript editing. SDH: Involved in manuscript editing. HB: Involved in manuscript writing‐up and editing. GA: The conception and manuscript writing‐up.

## FUNDING INFORMATION

None.

## CONFLICT OF INTEREST STATEMENT

The authors declared that no conflict of interest exist for this work.

## Data Availability

The data presented in this manuscript are available from corresponding author upon reasonable request.
